# Detection and quantification of synthetic cannabinoids in seven illicitly sourced disposable vapes submitted by an individual presenting to a UK drug and alcohol service

**DOI:** 10.1111/add.16671

**Published:** 2024-09-10

**Authors:** Sam Craft, Peter Sunderland, Molly F. Millea, Christopher R. Pudney, Oliver B. Sutcliffe, Tom P. Freeman

**Affiliations:** ^1^ Addiction and Mental Health Group, Department of Psychology University of Bath Bath UK; ^2^ Department of Life Sciences University of Bath Bath UK; ^3^ MANchester DRug Analysis and Knowledge Exchange (MANDRAKE) Manchester Metropolitan University Manchester UK

**Keywords:** Cannabis, disposable vapes, HHC, semisynthetic cannabinoids, synthetic cannabinoids, THC‐O, vaping

## Abstract

**Background and aims:**

In the United Kingdom and internationally, synthetic cannabinoids (SCs) are a common adulterant in illicitly sourced vaping products. Recently, their use is increasingly being linked to severe health effects, particularly among children. Here, we aimed to conduct the first detection and quantification of SCs in illicit disposable vaping products.

**Methods:**

A cross‐section of seven illicitly sourced disposable vape samples that were initially sold as cannabis products was submitted for analysis by a single individual presenting to a drug and alcohol service in the United Kingdom. Qualitative and quantitative analyses of these samples were conducted using nuclear magnetic resonance and gas chromatography/electron ionization‐mass spectrometry.

**Results:**

Qualitative analysis identified the SC 5F‐MDMB‐PICA in all seven samples, in the absence of any other pharmacologically active compounds. Quantitative analysis revealed that the median concentration of 5F‐MDMB‐PICA was 0.85 mg/ml (range = 0.59–1.63). The external appearance of these vape samples closely resembled regulated vaping products, and the presence of SCs was not identifiable by any labelling or packaging.

**Conclusions:**

The SC 5F‐MDMB‐PICA was detected at a median concentration of 0.85 mg/ml in seven disposable vapes which were illegally sourced in the United Kingdom, were mis‐sold as cannabis products and closely resembled legal, regulated products.

## INTRODUCTION

During recent years, the use of electronic cigarettes (e‐cigarettes) or vapes have seen a rapid increase in popularity around the world. While their effectiveness as a smoking cessation tool is now well established [1], they have also evolved into diverse consumer products which are used outside that context. In particular, the use of disposable vapes—which retail at lower prices and are available in a variety of different flavours—has increased use among those not previously smoking, especially among children and adolescents [2, 3]. Additionally, vapes are now being used as a delivery device for a broad range of illicit drugs [4] and in particular have become a common way of consuming cannabis in both legal and illicit markets [5].

Although the harms and long‐term effects of vaping are poorly understood, the use of unregulated or illicitly sourced vaping products has become a major safety risk. Most notably, illicit cannabis vaping products containing vitamin E acetate were primarily responsible for the e‐cigarette or vaping product use‐associated lung injury (EVALI) outbreak in which more than 2800 patients were hospitalized in the United States between August 2019 and February 2020. EVALI cases have since sharply declined; however, together with the rise in legal sales of disposable vapes, there has also been a rapid expansion in the illicit market for these products. For example, in the United Kingdom more than 2.5 million illicit vapes have been seized since 2020 [6], and according to the National Trading Standards Institute they now represent one of the largest consumer protection threats. These illicit products closely resemble legal, regulated products and they may be sold in the same outlets [6]; however, the lack of regulatory control introduces various safety risks. For example, non‐compliant electrical components may pose fire hazards, nicotine contents often exceed those permitted by law and they may contain constituents which are harmful to human health.

In the United Kingdom and internationally, illicit e‐liquids marketed as cannabis products have become a common source of drug contamination, particularly with synthetic cannabinoids (SCs) [7, 8]. For example, a report from the UK drug‐checking service WEDINOS revealed that SCs were present in 46% of all e‐liquids submitted to the service between 2020 and 2021, and the majority of these were purchased as cannabis‐based products [7]. SCs are a broad class of drugs which are notionally designed to mimic the effects of delta‐9‐tetrahydrocannabinol (THC), the main psychoactive component of cannabis. Unlike THC, which acts as a partial agonist at cannabinoid (CB1 and CB2) receptors, SCs typically act as potent full receptor agonists and as a result have a higher risk of severe toxic effects, including cardiorespiratory complications and seizures [9]. Recently there have been increasing reports in the UK media of SCs appearing in vaping products which have caused severe health effects, particularly among children while in attendance at school [10]. Furthermore, between 1997 and 2020 only two deaths associated with vaping products have been reported in the United Kingdom, both of which involved the use of SCs [11].

However, despite the increasing trend towards the use of disposable vapes and the growing illicit market for these products, SCs have so far only been identified in e‐liquids or re‐usable devices. The contamination of harmful illicit drugs in disposable vaping products could represent an important shift in the drugs market and a new consumer threat. Here we aim to report, to our knowledge, the first ever detection and quantification of SCs in sealed disposable vapes.

## METHODS

### Sample

**FIGURE 1:**
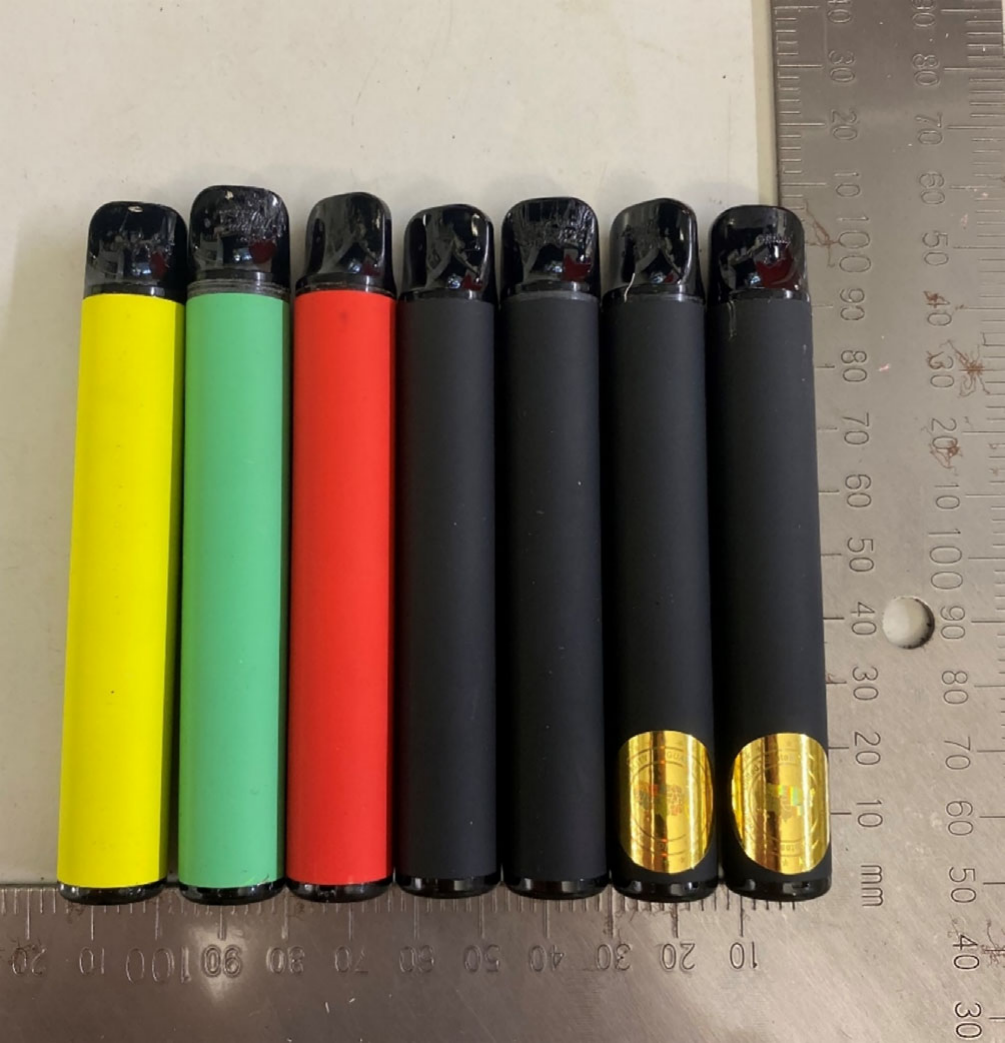
The seven unlabelled disposable vape samples volunteered for analysis (from left to right: samples 1–7).

A cross‐section of seven sealed and unlabelled disposable vapes (see Figure 1) was volunteered by a single individual presenting to a drug and alcohol service in the United Kingdom. These samples were received via an established partnership between this service and the University of Bath which, with approval and support from the Avon and Somerset Police, enables drugs of potential interest to be transferred for analysis.

### Analysis

A detailed description of the analytical methods is reported in the Supporting information. Briefly, qualitative analyses were conducted by nuclear magnetic resonance (NMR) and quantitative analysis by gas chromatography electron ionization‐mass spectrometry (GC‐EI‐MS). Quantitative analyses were conducted in triplicate and data presented represent the mean concentration for the three replicates of each sample together with the relative standard deviation (% RSD) expressed as an upper/lower limit of the mean. Analyses were not pre‐registered and results should be considered exploratory.

## RESULTS

### Self‐reported details of use and adverse effects

**FIGURE 2:**
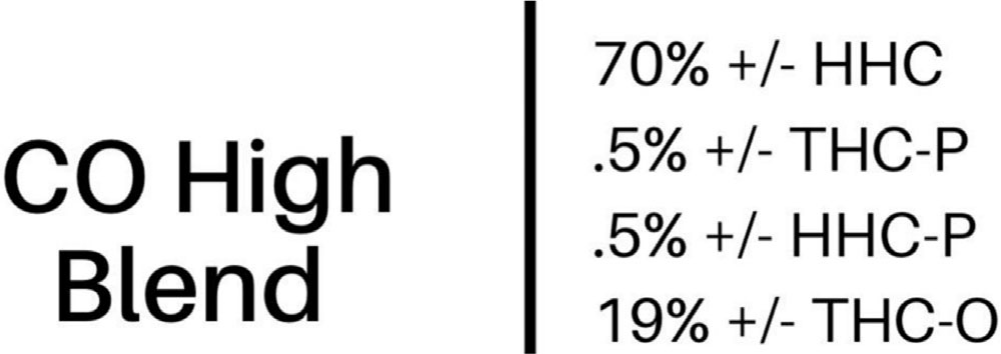
The certificate of analysis (COA) provided by the supplier of the unlabelled disposable vapes detailing the purported composition of these products. HHC = hexahydrocannabinol; THC‐P = tetrahydrocannabiphorol; HHC‐P = hexahydrocannabiphorol; THC‐O = tetrahydrocannabinol acetate ester. ‘CO High Blend’ appears to be the name given to these products, which potentially reflects the mixture of different semisynthetic cannabinoids.

The service user was a 45‐year‐old white male in full‐time employment and living in privately owned accommodation. All samples were purchased illicitly from the same UK‐based supplier who was purportedly sourcing the products from the United States. They were initially sold as ‘THC‐based products’; however, the supplier later provided a certificate of analysis (COA) which reported the contents as a mixture of the following four semi‐synthetic cannabinoids: hexahydrocannabinol (HHC), hexahydrocannabiphorol (HHC‐P), tetrahydrocannabiphorol (THC‐P) and the tetrahydrocannabinol acetate ester (THC‐O; see Figure 2).

After initially using these products in small quantities (i.e. ‘one or two puffs a few times a day’), the service user described the effects to be ‘strong but enjoyable’ and noted that the vapes allowed for more discreet use (compared to smoking herbal material). His use then rapidly escalated and he began using a single vape to completion every day for approximately 1 month. During this period, he reported experiencing a range of adverse effects including stomach cramps, loose bowels, loss of memory, difficulty focusing and feelings of dissociation from reality. He also reported experiencing a range of additional symptoms upon cessation, including extreme anxiety, panic attacks, low and angry moods, irritability and chest pains.

The service user reported regular cannabis use within the past year (once or twice a week) but no previous history of SC use.

### Qualitative and quantitative analysis

**FIGURE 3:**
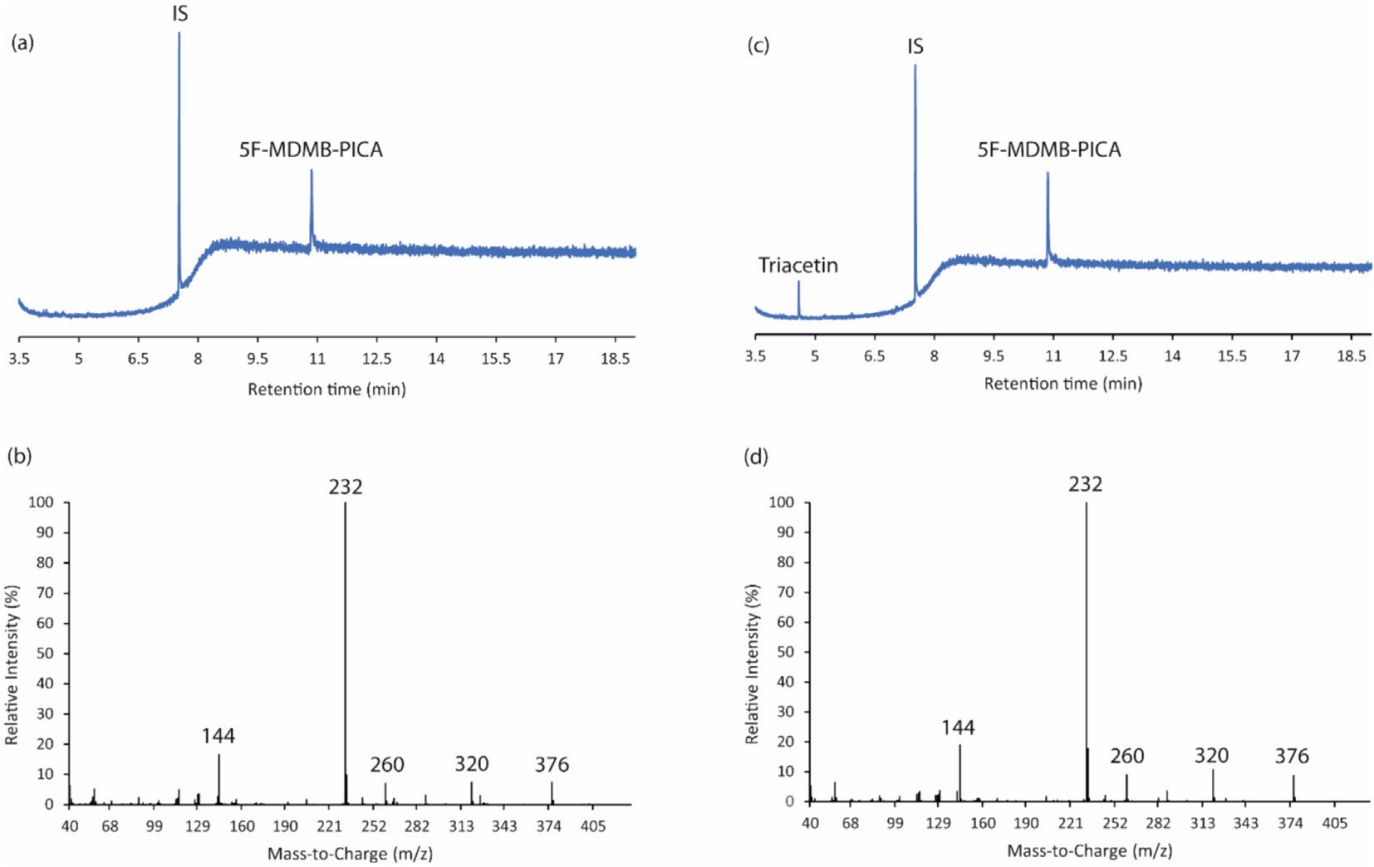
(a) Representative total ion chromatogram of 5F‐MDMB‐PICA (1 mg/ml) containing methyl stearate (IS, 70 μg/ml) in methanol; (b) EI‐MS spectrum (+ve ion mode) of 5F‐MDMB‐PICA (t_R_ = 10.86 minutes) standard; (c) representative total ion chromatogram of sample 2 containing methyl stearate (IS, 70 μg/ml); (d) EI‐MS spectrum (+ve ion mode) of sample 2 (t_R_ = 10.86 minutes).

NMR analysis revealed the presence of 5F‐MDMB‐PICA [in addition to water, ethanol, propylene glycol (PG) and vegetable glycerine (VG); see Supporting information, Figure S2 for an example NMR spectrum] in each of the seven samples. The GC‐EI‐MS analysis also corroborated that each of the seven samples contained 5F‐MDMB‐PICA as the major component (t_R_ = 10.86 minutes) together with triacetin as a minor component (t_R_ = 4.57 minutes). An exemplar GC‐EI‐MS total ion chromatogram of one sample (sample 2) and the corresponding electron ionization (EI) mass spectra are presented in Figure 3c,d, together with those of the reference standard of 5F‐MDMB‐PICA (Figure 3a,b).

The results of the quantitative analyses, which are summarized in Table 1, showed that the median concentration of 5F‐MDMB‐PICA among all samples was 0.85 mg/ml (range = 0.59–1.63), equivalent to a median of 0.08% (range = 0.05–0.14) of the samples’ total weight.

**TABLE 1:** Results of the qualitative and quantitative gas chromatography electron ionization‐mass spectrometry (GC‐EI‐MS) analyses of the seven unlabelled disposable vape samples. Concentrations refer to mean values for the replicates of each sample ± relative standard deviation.

Sample	Weight of extract (g)	Qualitative analysis	Quantitative analysis (mg/ml)	Quantitative analysis (%w/w)
1	1.14	5F‐MDMB‐PICA	0.85 ± 0.19	0.08 ± 0.02
2	1.14	5F‐MDMB‐PICA	1.16 ± 0.17	0.10 ± 0.02
3	1.14	5F‐MDMB‐PICA	0.59 ± 0.20	0.05 ± 0.02
4	1.03	5F‐MDMB‐PICA	0.67 ± 0.05	0.06 ± 0.004
5	1.10	5F‐MDMB‐PICA	0.95 ± 0.19	0.08 ± 0.02
6	1.12	5F‐MDMB‐PICA	1.63 ± 0.04	0.14 ± 0.004
7	1.35	5F‐MDMB‐PICA	0.78 ± 0.13	0.07 ± 0.01
Sample total (median; range)	1.14 (1.03–1.35)	–	0.85 (0.59–1.63)	0.08 (0.05–0.14)

Abbreviations: g = grams; mg = milligrams; ml = millilitres; w/w = weight by weight.

Externally, the vape devices varied in colour (black, yellow, green and red) and their appearance closely resembled legal regulated products. No samples contained any identifiable labels or packaging describing their contents; however, samples 6 and 7 (see Figure 1) both contained a tamper‐evident label bearing the inscription ‘Product of the United States’ which after removal created a void text mark.

## DISCUSSION

The contamination of SCs in vaping products is an emerging public health issue that is increasingly resulting in reports of adverse health effects [10]. Here, we report the detection and quantification of the SC 5F‐MDMB‐PICA in unlabelled disposable vapes that were volunteered by an individual presenting to a drug and alcohol service after experiencing various adverse effects. Since 2019, 5F‐MDMB‐PICA has been one of the most commonly detected SC compounds in the United States and in the United Kingdom [12, 13] and it has been implicated in several fatal and non‐fatal poisonings [13, 14].

Quantitative analyses of the vape samples revealed that the median concentrations of 5F‐MDMB‐PICA in these products was 0.85 mg/ml (range = 0.59–1.63). Although existing data on the concentration of SCs encountered in vaping products is sparse, this concentration range is similar to those reported in a previously published analysis of e‐liquid samples seized in China [15]. Disposable vapes typically have an e‐liquid capacity of 2 ml, meaning that if used to completion, a single vape containing SCs at the concentrations identified here would expose users to approximately 2 mg of SC material. By contrast, the median concentration of SCs in the most recently published analysis of herbal material was 13 mg per gram [16], and in a large international survey SC users reported using an average of 0.5 g per session [17]. However, despite the low concentration, the service user submitting the vapes analyzed here reported experiencing a range of adverse effects, including symptoms consistent with SC withdrawal (e.g. irritability, anxiety, low mood) upon cessation [17].

These products were initially sold as THC‐based products, although a COA later provided by the supplier reported the contents as a mixture of semi‐synthetic cannabinoids including HHC and THC‐O. Semi‐synthetic cannabinoids are newly emerging products which are reported to produce similar effects to THC and are often sold as legal alternatives to cannabis—especially in the United States [18], from where these samples were purportedly sourced. However, unlike the legal retail market for THC products in some US states, the sale of semi‐synthetic cannabinoid products remains unregulated and there are no quality or safety controls. There is therefore a risk that semi‐synthetic cannabinoid products may be mis‐sold as SCs more widely throughout the United States and elsewhere, and consumers should be aware that COAs may be falsified and should not be used to guarantee the contents (or safety) of products purchased on the illicit market.

E‐liquids marketed as THC or cannabis have become a common source of drug contamination, not just with SCs but also other illicit drugs, including most recently xylazine [19]. The vapes analyzed here were also sold as cannabis products; however, their appearance closely resembled regulated nicotine‐based disposable vapes and they could conceivably be sold or mistaken as such. It is not possible to determine the content of these products based on their external or internal appearance, and any products sourced illicitly are likely to pose significant risks of unintended drug exposure, regardless of marketing or purchase intent. Furthermore, governments in several different countries, including the United Kingdom and Australia, are beginning to introduce bans on the sale of disposable vaping products. Also, the United Kingdom has recently passed a law which will prevent anyone born after the year 2009 from ever legally purchasing tobacco products (although this law does not extend to re‐useable vaping devices or e‐liquids and other products containing nicotine) [20]. It is possible that restricting legal access to these products could lead to the expansion of the illicit market as consumers seek alternative sources, and this could further increase potential risks.

To help mitigate these risks, the potential harms of illicitly sourced products need to be effectively communicated to consumers, especially young people, who may be particularly likely to purchase vapes from illicit sources (e.g. from friends or dealers) due to age‐related sale restrictions of legal products [6]. Risks also need to be communicated to individuals working in schools and other environments where use of illicit vaping products may be more common—such as prison settings (due to the tobacco smoking ban and high rates of SC use within this population)—and measures to prevent their use should be implemented. Also, to improve treatment and diagnosis, health‐care professionals should be aware that adverse effects associated with vaping product use may be the result of acute drug toxicity.

Drug‐checking services can play a crucial role in monitoring and identifying potential risky trends in drug markets; however, the detection of drugs in vaping products and other complex matrices presents major technical challenges which prevent rapid, field‐based testing of these products [8]. This is an important limitation, as vapes are becoming a common way to consume a broad range of drugs [4] and, as shown here and elsewhere, they may be mis‐sold and adulterated with other potentially more harmful drugs [7, 8, 19]. Disposable vapes also present additional challenges as these products are sealed and cannot be used after their contents have been extracted for analysis, and they may be used by individuals who do not typically engage with drug‐checking or other harm reduction services. There is therefore a need for these services to adapt to the changing landscape of drug use and improve their capacity and reach to rapidly test drugs in vaping products.

This study has important strengths. As far as we are aware, this is the first study to conduct analyses of used and sealed disposable vaping products and the presence of SCs were confirmed via two validated methods (NMR and GC‐EI‐MS). However, it has important limitations. Analyses were conducted on a small sample of vapes volunteered by a single individual, which cannot be considered representative of the wider market for these products. Nonetheless, as the first study to identify and quantify SCs in disposable vaping products, this strongly warrants urgent investigation in the content of these products on a larger scale.

## CONCLUSION

Here we report the first study, to our knowledge, to detect and quantify SCs in illicitly sourced disposable vapes, volunteered by an individual after experiencing various adverse effects. The median concentration of SCs in the samples analysed here was 0.85 mg/ml and their appearance closely resembled legal regulated vaping products.

## AUTHOR CONTRIBUTIONS

**Sam Craft:** Conceptualization (lead); data curation (lead); formal analysis (equal); investigation (lead); methodology (equal); project administration (lead); writing—original draft (lead). **Peter Sunderland:** Conceptualization (equal); formal analysis (equal); methodology (equal); supervision (equal). **Molly F. Millea:** Formal analysis (equal); investigation (equal). **Christopher R. Pudney:** Funding acquisition (equal); supervision (equal). **Oliver B. Sutcliffe:** Formal analysis (equal); methodology (equal); supervision (equal). **Tom P. Freeman:** Conceptualization (equal); supervision (lead).

## DECLARATION OF INTERESTS

None to declare.

## Supporting information


**Figure S1.** A dismantled vape and the internal components from which residual e‐liquid was extracted for analysis.
**Figure S2.** Exemplar ^1^H NMR spectrum (500 M Hz in MeOD) displaying the presence of 5F‐MDMB‐PICA, water, ethanol, propylene glycol and glycerol in sample 2.

## Data Availability

The data that support the findings of this study are available from the corresponding author upon reasonable request.
