# Supplementary material for: Detection and quantification of synthetic cannabinoids in seven illicitly sourced disposable vapes submitted by an individual presenting to a UK drug and alcohol service
Source: Addiction. 2024 Sep 10;120(3):549–54. doi: 10.1111/add.16671 (PMC11813733; doi:10.1111/add.16671)
Supplement: Supplementary file 1 — Figure S1. A dismantled vape and the internal components from which residual e‐liquid was extracted for analysis. Figure S2. Exemplar 1H NMR spectrum (500 M Hz in MeOD) displaying the presence of 5F‐MDMB‐PICA, water, ethanol, propylene glycol and glycerol in sample 2. [file ADD-120-549-s001.docx]

**Supplementary Materials**

*Materials*

All sovents were HPLC grade (> 99.8% purity) and purchased from Merck UK. The 5F-MDMB-PICA standard was purchased from LGC Standards UK (> 99.8% purity).

*Extraction*

All vape samples had been used to completion; however, residual e-liquid could be extracted from internal components after each device was manually dismantled. The absorbent pad, mouth-piece and all other internal components (see Figure S1) were submerged in 50 mL of methanol (MeOH) for 3 minutes. The MeOH was then removed, and this process was repeated 3 times to ensure full extraction of the e-liquid. After the final iteration, 50 µL of the methanolic extract was reserved for GC-EI-MS analysis, however, before conducting the NMR analysis we completed a further extraction on the remaining methanolic extract (following the same methods as above) using hexane. This enabled the removal of the majority of propylene glycol (PG) and glycerol (VG) from the sample and therefore allowed cleaner NMR spectra.

*Nuclear Magnetic Resonance (NMR) Spectroscopy*

Qualitative analysis was completed by ^1^H NMR. NMR spectra were recorded in Methanol D4 on a Bruker AVANCE III 500 MHz spectrometer.

*Gas Chromotography-Electron Ion-Mass Spectrometry (GC-EI-MS)*

GC-EI-MS analysis was performed using an Agilent 7890B GC and a MS5977B mass selective detector (Agilent Technologies, Wokingham, UK). The mass spectrometer was operated in the electron ionisation mode at 70 eV. Separation was achieved with a capillary column (HP-5MS, 30 m length, 0.25 mm i.d., 0.25 μm film thickness) with helium as the carrier gas at a constant flow rate of 1.2 mL/min. The following oven temperature program was used: 50 to 290 °C at 30 °C/min, hold for 8 min for a 16 min total runtime. A 2 μL injection volume was used, with a split ratio of 50:1. The injector and the GC interface temperatures were both maintained at 280 °C, respectively. The MS source and quadrupole temperatures were set at 230 °C and 150 °C. All standards and test samples were prepared as solutions, in methanol with no derivatisation. Methyl stearate (70 µg/mL) was used as an internal standard and each standard/test sample was injected in triplicate. Mass spectra were obtained in full scan mode (*m*/*z* = 40–550) for 5F-MDMB-PICA (t_R_ = 10.86 min) and methyl stearate (t_R_ = 7.53 min), respectively. The spectral data was compared to a standard of 5F-MDMB-PICA (prepared in-house using the method reported by Antonides *et al.* (1) and this is presented in Figure 3a/b. The mean concentration for the three replicates of each sample were calculated along with the relative standard deviation (% RDS) expressed as an upper/lower limit of the mean.

**
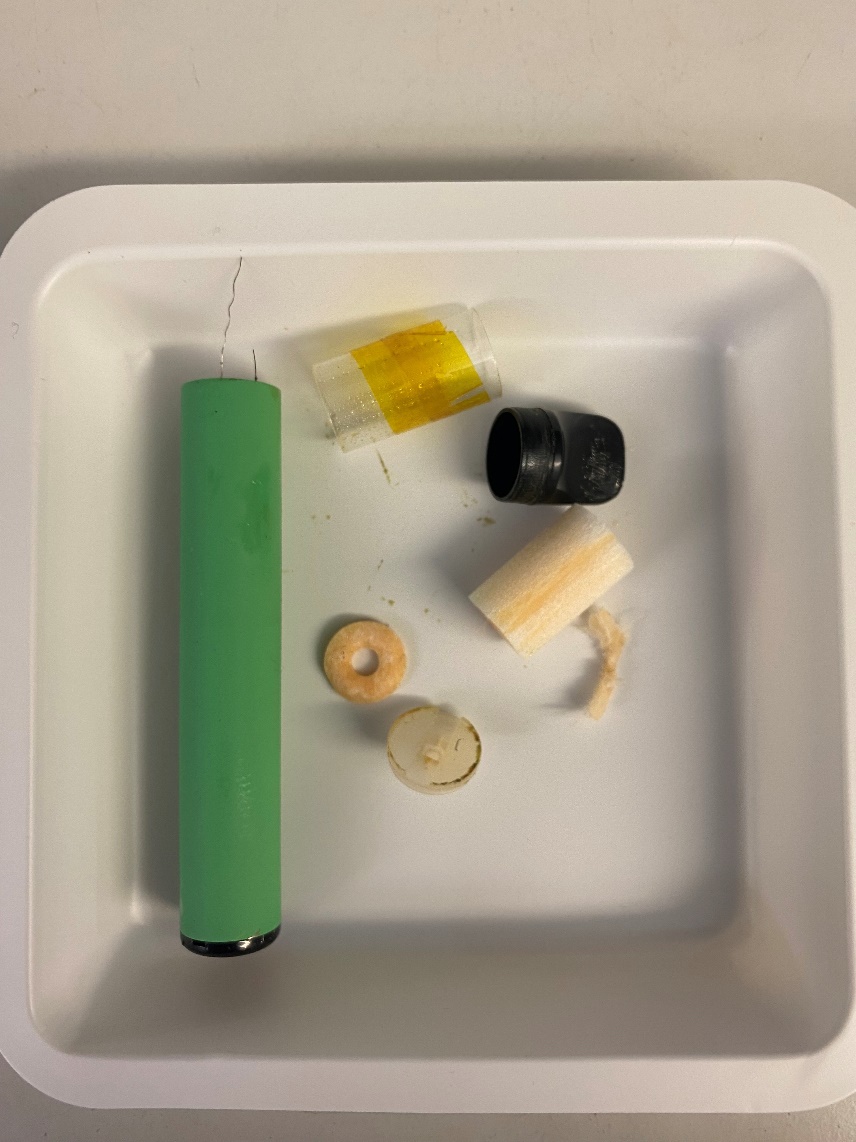
Figure S1.** A dismantled vape and the internal components from which residual e-liquid was extracted for analysis.


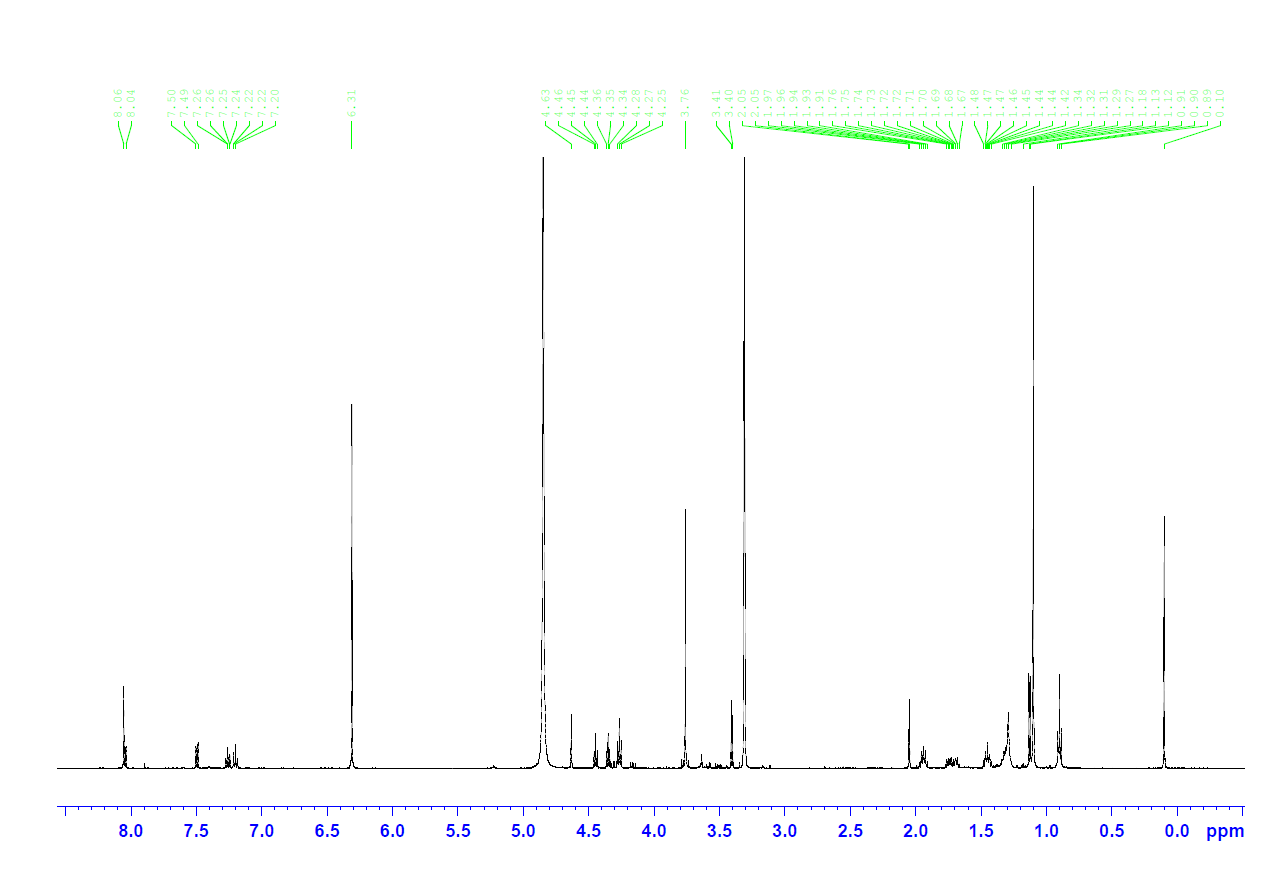
**Figure S2.** Exemplar ^1^H NMR spectrum (500 M Hz in MeOD) displaying the presence of 5F-MDMB-PICA, water, ethanol, propylene glycol and glycerol in sample 2

**References**

Antonides LH, Cannaert A, Norman C, NicDáeid N, Sutcliffe OB, Stove CP, et al. Shape matters: The application of activity-based in vitro bioassays and chiral profiling to the pharmacological evaluation of synthetic cannabinoid receptor agonists in drug-infused papers seized in prisons. Drug Test Anal. 2021;13(3):628-43.
